# The placement of four different supraglottic airway devices by medical students: a manikin study

**DOI:** 10.1080/07853890.2023.2282746

**Published:** 2023-11-20

**Authors:** Keziban Bollucuoğlu, Çağdaş Baytar, Gamze Küçükosman, Hilal Ayoğlu

**Affiliations:** Department of Anesthesiology and Reanimation, Zonguldak Bülent Ecevit University Medicine Faculty, Zonguldak, Turkey

**Keywords:** Supraglottic airway device, medical students, manikin study, medical education, airway management

## Abstract

**Background and objectives:**

Although endotracheal intubation is the gold standard in airway management, this procedure requires both technical training and experience. Supraglottic airway devices are an alternative to endotracheal intubation and are simpler, less invazive, and require less time for placement compared with endotrakeal intubation. Aim of the study was to evaluate the success rates, ease of use, duration of application, and maneuver performance of different supraglottic airway devices (SADs) used by term-5 medical students on a manikin.

**Materials and methods:**

This cross-sectional study was conducted in Zonguldak Bülent Ecevit University Hospital, Turkey, between April and June 2022. Term 5 Medical students (*n* = 111) were asked to place four different SAD [classical laryngeal mask, suprema laryngeal mask, ProSeal laryngeal mask (pLMA), I-gel] on an adult airway manikin. After the students were trained in the use of the devices, the ease of use for each, duration of successful application, success of application and use of optimization maneuvers were recorded. The participants were asked to distinguish the device they felt most confident to place and the most difficult to implement.

**Results:**

There was a significant difference between the groups in ease and duration of application (*p* < 0.001). The most difficult and longest application time was with pLMA and the easiest and shortest was with I-gel (*p* < 0.05). The number of application failure was also highest for pLMA (*p* < 0.001). It was found that the participants distinguished (41%) I-gel as the most confident device to use, (84%) pLMA as the most difficult device to use for airway control.

**Conclusions:**

I-gel was found to be superior to others in terms of ease of use, duration and success of application.

## Introduction

**Figure 1.**
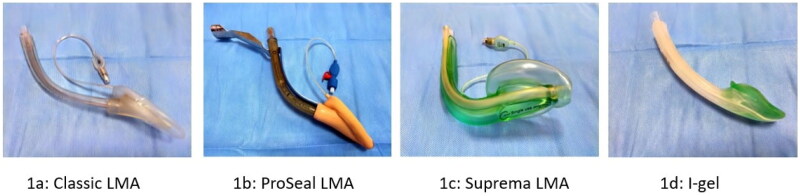
Supraglottic airway devices.

Endotracheal intubation (ETI) is the gold standard for airway management. However, because ETI should only be performed by professional healthcare providers with appropriate technical training and experience, the complication rate is quite high when performed by inexperienced medical personnel [1]. Therefore, supraglottic airway devices (SADs) are an alternative to ETI. These devices allow the patient to be ventilated without having to cross the glottis [2]. SAD placement have been reported to be simpler, less invasive, and require less time for placement compared with ETI [3,4]. To date, many airway devices have been manufactured. The classic laryngeal mask airway (cLMA), which was first introduced, has disadvantages such as gastric air filling and airway leakage during positive pressure ventilation [5,6] (Figure 1(a)). The ProSeal laryngeal mask (pLMA) is a device with a larger wedge-shaped cuff and a bite block, and it provides effective ventilation because the cuff prevents air leak at high pressure. There are four different placement methods: using the index finger, by slightly inflating the cuff, as if inserting an airway and with the help of an applicator. One of its important feature is the drainage tube that allows aspiration of gastric contents [6] (Figure 1(b)). The suprema laryngeal mask (sLMA), a second-generation SAD, is a laryngeal mask with a polyvinyl chloride cuff that allows aspiration of gastric contents. Its oblique structure allows ease of placement and the gastric channel allows gastric drainage [3] (Figure 1(c)). The I-gel, on the other hand, is a new generation SAD that has no cuff, expands with body temperature due to its thermoplastic elastomer structure, and fully conforms to the supraglottic tissue, aims to minimize air leakage. In addition, the presence of a gastric drainage tube that allows access to stomach *via* a nasogastric catheter reduces the risk of aspiration of gastric contents into the lungs [7,8] (Figure 1(d)).

Since airway management is a life-saving procedure, all healthcare professionals, including medical students, should be taught this practice. While it has been reported that inexperienced healthcare professionals are easier to train in the use of laryngeal mask in airway management (LMA) than ETI, there is an accepted opinion that students should be trained in airway management by using different airway device on simulators before allowing direct contact with patients.

There are many studies comparing the success and duration of application on the manikin by different SAD with non-professionals (paramedics, medical students, non-anesthesiologists, and medical assistants) [8–15]. Airway management training is one of the components taught to medical students in our country. In our study, we aimed to compare the success rate, ease and duration of application, and maneuver use in cLMA, sLMA, pLMA and I-gel applications on a manikin by term 5 medical students undertaking an anesthesia internship in Turkish medicine faculty.

## Materials and methods

Our cross-sectional study was conducted between April and June 2022 after obtaining Local Ethics Committee approval (reference number: 2022/03-13, ClinicalTrials.gov identifier: NCT05523752) and written informed consent from the participants.

We planned to include 111 term 5 students who participated in the Anesthesiology and Reanimation internship at Zonguldak Bülent Ecevit University medicine faculty and agreed to participate in the study. The students were informed about the study at an information meeting on the day they started their internship. The students who were informed about the study before the study onset were asked to not share the information to avoid any bias in the study. Those who had already used four different SAD, had received airway management training, and were unwilling to participate in the study were excluded.

Participants received theoretical training on airway anatomy and physiology as well as basic and advanced airway management by a lecturer in the Department of Anesthesiology and Reanimation. Following this training, the lecturer provided instructions for use and demonstrations for each SAD, including the information in the manufacturer’s manuals for each, with a 5-min video and a 15-min presentation.

**Figure 2.**
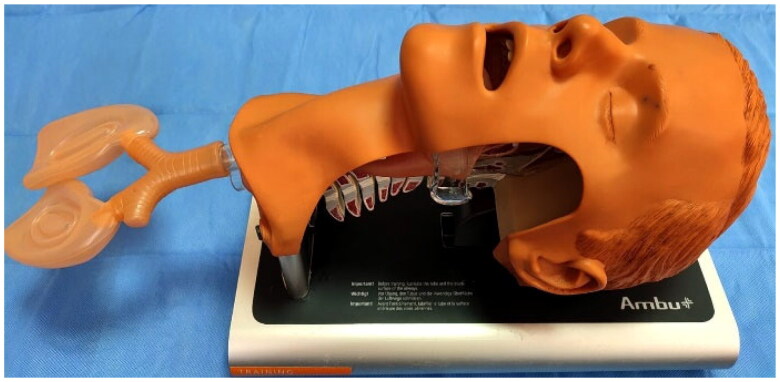
Adult airway manikin.

The manikin used was a standard adult airway manikin (Ambu^®^ Airway Management Trainer, USA) (Figure 2) that has been proven to work with various supraglottic devices, and the devices were placed on the manikin in the “sniffing position” (head tilted, chin raised, and neck flexed) [9]. Supraglottic airway devices used in this study; cLMA (Tuoren, Changyuan, Henan, China™), sLMA (Glen Burnie USA™), pLMA (San Diego, USA™), and I-gel (Intersurgical Ltd., Wokingham, UK™). Materials to be used during application, such as lubricant and syringe, were kept ready next to the airway manikin. Before the start of the study, all available adult-sized SADs were tried on the manikin by an anesthesiologist blinded to the study to determine the proper size of the devices. It was found that the size 4 SAD was suitable. After application the cuffed devices were inflated to the recommended cuff volume to fit the manikin’s larynx.

The number of previous placement attempts required for a healthcare professional to achieve a successful LMA placement in a manikin has been reported to be between three and eight [10]. All participants were asked to practice on the manikin by placing the SAD; used in the study under the guidance of the same senior anesthesiologist three consecutive times in the same order (respectively; cLMA, sLMA, pLMA, I-gel). The student used the applicator method for pLMA. During the fourth placement of each SAD, the duration of application (the duration between the participants taking the supraglottic airway devices in their hands and the confirmation of complete placement in the airway with the ambu mask), ease of use (0, easiest; 100, most difficult), success of application (failure of the manikin’s lungs to ventilate within 30 s with the ambu mask was considered unsuccessful), and whether maneuvers were performed during placement (head extension and rotation) were evaluated and recorded by the anesthesiologist (ÇB). A stopwatch was used to determine application times. To avoid any learning effects during the whole process, participants were not allowed to watch each other during the placement attempts. After all procedures, the participants were asked to answer the following questions:

1. Which SAD do you feel most confident in using to maintain airway control?
2. Which SAD do you think is the most difficult to use for achieving airway control?

All data were recorded on the designed form by the same anaesthetist (ÇB).

### Statistical analysis

The Statistical Package for the Social Sciences (SPSS) 22 program was used for data analysis. Data are presented as number, percentage, mean, and standard deviation. Kolmogorov Smirnov test was used to assess normal distribution. Parametric tests were used to analyze the data that fit the normal distribution and non-parametric tests were used to analyze the data that did not. Kruskal-Wallis and Chi-square tests were used for group analysis. *p* < 0.05 was considered significant.

## Results

A total of 111 term 5 medical students, 57 (51.3%) males and 54 (48.7%) females, with a mean age of 23.71 ± 1.27 years participated in the study. No student refused to participate in the study.

A statistically significant difference was found between the groups in terms of ease of use and duration of application (*p* < 0.05; Table 1). Regarding application, I-gel was rated as the easiest and pLMA as the most difficult; similarly, the application time was shortest for I-gel and longest for pLMA.

**Table 1.** Comparison of ease of use and duration of application according to groups.

	cLMA	sLMA	pLMA	I-gel
Ease of Use	29.3 ± 19.1	27.61 ± 8.1	57.4 ± 27.2^&^	21.2 ± 19.9*
Duration of application (sec)	15.6 ± 13	13.7 ± 9.7	21 ± 8.4**^∞^**	10.3 ± 3.7**^#^**

cLMA: Classic laryngeal mask airway; sLMA: Suprema laryngeal mask airway; pLMA: ProSeal laryngeal mask airway.

Kruskal-Wallis test.

**POST-HOC of EASE OF USE**.

*Compared I-gel with cLMA, sLMA, pLMA (respectively *p* < 0.005, <0.038, *p* < 0.001).

^&^Compared pLMA with cLMA, sLMA (respectively *p* < 0.001, *p* < 0.001).

**POST-HOC of DURATION OF APPLICATION**.

**^#^**Compared I-gel with cLMA, sLMA, pLMA (respectively *p* < 0.001, *p* < 0.001, *p* < 0.001).

**^∞^**Compared pLMA with cLMA, sLMA (respectively *p* < 0.001, *p* < 0.001).

There was a statistical difference between the groups in terms of application success, and the failure rate was highest when pLMA was used (*p* < 0.001). It was observed that there was a statistically significant difference between the groups regarding the use of maneuvers, which was higher in the pLMA application (*p* < 0.001; Table 2).

**Table 2.** Comparison of success of application and use of maneuvers according to groups.

	cLMA *n* (%)	sLMA *n* (%)	pLMA *n* (%)	I-gel *n* (%)	*p*
Success	105 (94.6)	109 (98.2)	89* (80.2)	110 (99.1)	<**0.001**
Maneuvers	58^a^ (52)	40^b^ (36)	81^c^ (73)	47^a,b^ (42)	<**0.001**

cLMA: Classic laryngeal mask airway; sLMA: Suprema laryngeal mask airway; pLMA: ProSeal laryngeal mask airway.

Pearson Chi-square analysis.

*Comparation of pLMA with cLMA, sLMA, and I-gel.

^a–c^There is no difference between times with the same letter in a group.

At the end of the procedure, the participants answered the question: with which SAD did you feel the most confident in maintaining airway control, answered respectively; 40.5% I-gel, 34.2% sLMA, 24.3% cLMA, 0.9% pLMA. In the other question, 83.8% of the participants distinguished pLMA as the most difficult device to apply.

## Discussion

In our study, which was conducted with the participation of term five medical students who had not been previously trained in airway management, we concluded that all four different SADs could be placed successfully on the airway exercise manikin after brief training by the students. That students stated that they were more confident when placing the I-gel compared to other SADs. We also found that the I-gel was placed faster by the students.

Supraglottic airway devices have been shown to be easy and safe to use after a short training period [8,11–15]. Various clinical and model studies have reported that I-gel insertion by paramedic students is easy [13]. Kannaujia et al. [8] reported that I-gel was much easier to apply in a manikin study in which paramedic and medical students compared sLMA and I-gel. In two different studies using I-gel and sLMA, both devices were reported to be easy to use by inexperienced medical personnel on both practice manikin and anesthetized patients [14,15]. Our results are consistent with the literature.

Stroumpoulis et al. [11] compared I-gel and cLMA application in airway control on a practice manikin with experienced and inexperienced physicians. They reported that cLMA application time was shorter for experienced physicians (13.7 s) than that for inexperienced physicians (22 s); similarly, I-gel application time was shorter for experienced physicians (11.2 s) than that for inexperienced physicians (15.2 s). Because the difference between the two groups in I-gel application was quite small, inexperienced physicians might prefer I-gel to safely secure the airway. Castle et al. [13] reported that this time (12.3, 33.8, and 22.4 s, respectively) was the shortest for I-gel in their manikin study in which they compared the application times of three different SADs (I-gel, cLMA, and or) by paramedics.Gatvard et al. [16] found that the placement time for cLMA was 14.50 ± 1.8 s and 14.17 ± 0.6 s for pLMA, while this time was 7.2 ± 0.5 s for I-gel in cardiopulmonary resuscitation in a manikin study including professionals. They reported that I-gel reduced the time to ensure airway safety by 50% compared to cLMA and pLMA. In their manikin study comparing I-gel, laryngeal tube, and ETI, Nakstad et al. [17] showed that 80% of experienced physicians were able to secure the airway with I-gel in a mean time of 12.3 ± 3.6 s. Similarly, in our study, after a short training, I-gel application time was recorded to be the shortest (10.3 ± 3.7 s) and pLMA application time was the longest (21 ± 8.4 s). We consider that the lack of a cuff for I-gel may have affected the placement time as no additional procedure was needed to regulate the cuff pressure.

After a short training, Gupta et al. [18] compared the placement of I-gel and pLMA on a collared manikin in three positions (head end free (Group A), head against the wall (Group B), and sitting (Group C)) by 35 paramedics and reported that the success rate with I-gel was significantly higher than with pLMA (91% vs. 77% in Group A, 100% vs. 88% in Group B, and 100% vs. 74% in Group C). Similarly, in our study, the application failure rate was higher for pLMA (19.8%), and it was designated as the most difficult device to use. We attributed this to its larger, deeper, softer bowl, and non-linear anterior margin structure, and the need for the use of an applicator for application.

Gupta et al. [19] compared the performances of three different manikin-based SADs (I-gel, cLMA, pLMA) and reported that the maneuver usage required for optimal position was 27.5% for pLMA and 2.5% for cLMA, while no maneuver was required for I-gel. Similarly, in our study, it was found that there was a difference between the groups in terms of maneuver use, and the use of maneuver was higher in case of pLMA compared with cLMA and sLMA, and in case of cLMA compared with sLMA. This difference might be due to the difficulty of placing the pLMA on a manikin, although it is placed with an applicator, and the difference between cLMA and sLMA might be due to the L-anatomic shape of the sLMA, which allows it to be placed without the use of maneuvers.

### Limitation

Firstly, because the order of application of SADs was maintained and no interval was given between applications, and since the skills were acquired through repeated applications, laryngeal masks may need to be randomized during application in future studies. Secondly, this is a manikin study that does not reflect the clinical condition; thus, complications such as vomiting, intraoral injury, and sore throat could not be captured. Clinical studies are needed to ensure the validity of these data. Another limitation of the study is the application of devices designed to work with the warmth of the body to mould in using a manikin. The other limitation is that this study only included term 5 medical students who had completed the semester internship course in anesthesia and reanimation. A larger participation can be achieved with students from different classes and/or students from different medical schools to conduct a larger study.

## Conclusion

In SADs applications on a manikin by medical students who have not been trained in airway management; I-gel was found to be superior to others in terms of ease of use, duration and success of application.

## Data Availability

The data that support the findings of this study are available from the corresponding author, K.B., upon reasonable request.

## References

[1] Soar J, Böttiger BW, Carli P, et al. European resuscitation council guidelines 2021: adult advanced life ­support. Resuscitation. 2021;161:1–5. doi:10.1016/j.resuscitation.2021.02.010. 33773825

[2] Soar J, Nolan JP, Böttiger BW, et al. European Resuscitation Council guidelines for resuscitation 2015: section 3. Adult advanced life support. Resuscitation. 2015;95:100–147. doi:10.1016/j.resuscitation.2015.07.016. 26477701

[3] Gruber C, Nabecker S, Wohlfarth P, et al. Evaluation of airway management associated hands-off time during cardiopulmonary resuscitation: a randomised manikin follow-up study. Scand J Trauma Resusc Emerg Med. 2013;21(21):10. doi:10.1186/1757-7241-21-10. 23433462 PMC3598524

[4] Robak O, Leonardelli M, Zedtwitz-Liebenstein K, et al. Feasibility and speed of insertion of seven supraglottic airway devices under simulated airway conditions. CJEM. 2012;14(6):330–334. doi:10.2310/8000.2012.120658. 23131479

[5] Brain AI. The development of the Laryngeal Mask–a brief history of the invention, early clinical studies and experimental work from which the Laryngeal Mask evolved. Eur J Anaesthesiol Suppl. 1991;4:5–17. 1879414

[6] Natalini G, Lanza G, Rosano A, et al. Standard Laryngeal Mask Airway and LMA-ProSeal during laparoscopic surgery. J Clin Anesth. 2003;15(6):428–432. doi:10.1016/s0952-8180(03)00085-0. 14652119

[7] Jeon WJ, Cho SY, Baek SJ, et al. Comparison of the Proseal LMA and intersurgical I-gel during gynecological laparoscopy. Korean J Anesthesiol. 2012;63(6):510–514. doi:10.4097/kjae.2012.63.6.510. 23277811 PMC3531529

[8] Kannaujia AM, Srivastava U, Singh T, et al. Evaluation of I-Gel™ versus classic LMA™ for airway management by paramedics and medical students: a manikin study. Anesth Essays Res. 2020;14(1):166–169. doi:10.4103/aer.AER_37_20. 32843812 PMC7428105

[9] Jackson KM, Cook TM. Evaluation of four airway trainingmanikins as patient simulators for the insertion of eight types of supraglottic airway devices. Anaesthesia. 2007;62(4):388–393. doi:10.1111/j.1365-2044.2007.04983.x. 17381577

[10] Wahlen BM, Roewer N, Lange M, et al. Tracheal intubation and alternative airway management devices used by healthcare professionals with different level of pre-existing skills: a manikin study. Anaesthesia. 2009;64(5):549–554. doi:10.1111/j.1365-2044.2008.05812.x. 19413826

[11] Stroumpoulis K, Isaia C, Bassiakou E, et al. Comparison of the i-gel and classic LMA insertion in manikins by experienced and novice physicians. Eur J Emerg Med. 2012;19(1):24–27. doi:10.1097/MEJ.0b013e3283474ab3. 21593672

[12] Lee DW, Kang MJ, Kim YH, et al. Performance of intubation with 4 different airway devices by unskilled rescuers: manikin study. Am J Emerg Med. 2015;33(5):691–696. doi:10.1016/j.ajem.2015.03.006. 25800412

[13] Castle N, Owen R, Hann M, et al. Assessment of the speed and ease of insertion of three supraglottic airway devices by paramedics: a manikin study. Emerg Med J. 2010;27(11):860–863. doi:10.1136/emj.2009.084343. 20515910

[14] Wharton NM, Gibbison B, Gabbott DA, et al. I-gel insertion by novices in manikins and patients. Anaesthesia. 2008;63(9):991–995. doi:10.1111/j.1365-2044.2008.05542.x. 18557971

[15] Howes BW, Wharton NM, Gibbison B, et al. LMA Supreme insertion by novices in manikins and patients. Anaesthesia. 2010;65(4):343–347. doi:10.1111/j.1365-2044.2010.06262.x. 20180796

[16] Gatward JJ, Thomas MJ, Nolan JP, et al. Effect of chest compressions on the time taken to insert airway devices in a manikin. Br J Anaesth. 2008;100(3):351–356. doi:10.1093/bja/aem364. 18158311

[17] Nakstad AR, Sandberg M. Airway management in simulated restricted access to a patient–can manikin-based studies provide relevant data? Scand J Trauma Resusc Emerg Med. 2011;19:36. doi:10.1186/1757-7241-19-36. 21668944 PMC3125355

[18] Gupta A, Kabi A, Gaur D. Assessment of success and ease of insertion of ProSeal™ laryngeal mask airway versus I-gel™ insertion by paramedics in simulated difficult airway using cervical collar in different positions in manikins. Anesth Essays Res. 2020;14(4):627–631. doi:10.4103/aer.AER_72_20. 34349332 PMC8294411

[19] Gupta B, Gupta S, Hijam B, et al. Comparison of three supraglottic airway devices for airway rescue in the prone position: a manikin-based study. J Emerg Trauma Shock. 2015;8(4):188–192. doi:10.4103/0974-2700.166589. 26604523 PMC4626934

